# Technologies for Monitoring Lifestyle Habits Related to Brain Health: A Systematic Review

**DOI:** 10.3390/s19194183

**Published:** 2019-09-26

**Authors:** Diego Moreno-Blanco, Javier Solana-Sánchez, Patricia Sánchez-González, Ignacio Oropesa, César Cáceres, Gabriele Cattaneo, Josep M. Tormos-Muñoz, David Bartrés-Faz, Álvaro Pascual-Leone, Enrique J. Gómez

**Affiliations:** 1 Biomedical Engineering and Telemedicine Centre, ETSI Telecomunicación, Center for Biomedical Technology, Universidad Politécnica de Madrid, 28040 Madrid, Spain; psanchez@gbt.tfo.upm.es (P.S.-G.); ioropesa@gbt.tfo.upm.es (I.O.); cesar.caceres@urjc.es (C.C.); egomez@gbt.tfo.upm.es (E.J.G.); 2 Institut Guttmann, Institut Universitari de Neurorehabilitació adscrit a la UAB, 08916 Badalona, Spain; jsolana@guttmann.com (J.S.-S.); lelecat3@gmail.com (G.C.); jmtormos@guttmann.com (J.M.T.-M.); dbartres@ub.edu (D.B.-F.); apleone@hsl.harvard.edu (Á.P.-L.); 3 Universitat Autònoma de Barcelona, 08193 Barcelona, Spain, and with Fundació Institut d’Investigació en Ciències de la Salut Germans Trias i Pujol, 08916 Badalona, Spain; 4 Centro de Investigación Biomédica en Red, Biomateriales y Nanomedicina (CIBER-BBN), 28029 Madrid, Spain; 5 ETSI Informática, Universidad Rey Juan Carlos, 28933 Madrid, Spain; 6 Institut d’Investigacions Biomèdiques August Pi i Sunyer, 08036 Barcelona, Spain; 7 Departament de Medicina, Facultat de Medicina i Ciències de la Salut, i Institut de Neurociències, Universitat de Barcelona, 08036 Barcelona, Spain; 8 Hinda and Arthur Marcus Institute for Aging Research and the Center for Memory Health, Hebrew SeniorLife, Department of Neurology, Harvard Medical School, Boston, MA 02131, USA

**Keywords:** adaptive systems, biomedical engineering, brain health, brain modeling, modeling, monitoring, review, remote monitoring, sensor systems, telemedicine

## Abstract

Brain health refers to the preservation of brain integrity and function optimized for an individual’s biological age. Several studies have demonstrated that our lifestyles habits impact our brain health and our cognitive and mental wellbeing. Monitoring such lifestyles is thus critical and mobile technologies are essential to enable such a goal. Three databases were selected to carry out the search. Then, a PRISMA and PICOTS based criteria for a more detailed review on the basis of monitoring lifestyle aspects were used to filter the publications. We identified 133 publications after removing duplicates. Fifteen were finally selected from our criteria. Many studies still use questionnaires as the only tool for monitoring and do not apply advanced analytic or AI approaches to fine-tune results. We anticipate a transformative boom in the near future developing and implementing solutions that are able to integrate, in a flexible and adaptable way, data from technologies and devices that users might already use. This will enable continuous monitoring of objective data to guide the personalized definition of lifestyle goals and data-driven coaching to offer the necessary support to ensure adherence and satisfaction.

## 1. Introduction

The increase in life expectancy is associated with a higher incidence and prevalence of highly disabling neurological and psychiatric illnesses [1]. However, while age is known to be the main risk factor, it is not enough to trigger the appearance of illnesses, nor the onset of cognitive deterioration. 

Brain health is defined by Cattaneo et al. [2] as “the development and preservation of optimal brain integrity and neural network functioning for a given age”. Brain function is not solely defined by genetics and age. Brain function evolves throughout life with environmental exposure, and experience, and is thus shaped by plasticity. Brain plasticity can be modulated, suppressing some brain changes and enhancing others to obtain a better functional result in a particular individual. Interventions based on healthy life habits can promote brain plasticity and thus achieve beneficial functional results [3,4]. Even though the mechanisms of plasticity change across the lifespan, plasticity remains a critical factor throughout life, and a healthy brain is a plastic brain. The malfunction of the mechanisms of plasticity is a major cause for the symptoms and disabilities of neurological and psychiatric diseases [4].

A longitudinal study monitoring cognitive function for eight years, found that nearly 30% of the elderly retain good memory and cognitive function into old age, many matching the performance of young individuals [5]. These observations highlight that while age is the main risk factor to develop neurological and psychiatric illnesses, cognitive decline and disability are not an obligatory consequence of aging. Therefore, it is important to identify the individual characteristics, including brain health mechanisms, that allow some to maintain an optimal cognitive function and mental wellbeing throughout life.

Anthropological, epidemiological, sociological and psychological studies [6,7,8] reveal that modifiable environmental factors and lifestyles have an important impact on an individual’s risk of developing brain diseases. These include the type and quality of the cognitive, physical, and social activities, sleep and eating habits, as well as personality features, beliefs and expectations. A review by Bamidis et al. [9] focused on studies that have investigated the effects of cognitive, physical, and multidomain interventions to promote brain health in adults. The authors concluded that interventions affecting more than one domain (for example, combining cognitive and physical interventions) have a greater impact on brain health than those addressing a single domain.

The Barcelona Brain Health Initiative (BBHI) [2] has defined a taxonomy of domains that may have an impact on brain health. BBHI is an ongoing prospective longitudinal study focused on identifying the determinants of brain health. The main objectives of BBHI are: (i) To characterize lifestyle, cognitive, behavioral and environmental markers related to a given individual’s cognitive and mental functions in middle to old age, (ii) to assess the biological determinants predictive of maintenance of brain health, and (iii) to evaluate the impact of a controlled multi-dimensional lifestyle intervention on improving and maintaining brain health. The factors identified by the BBHI and hypothesized to be related to brain health are:

1. Physical exercise: The regular practice of physical exercise has been shown to have a deep impact on mood and stress tolerance, improving depression and anxiety. In addition, physical activity can improve cognitive function and improve wellbeing in a number of neurodegenerative diseases. It also has been repeatedly associated with the upregulation of neurotrophic factors. Different studies have linked being active with a lower prevalence of neurological and psychiatric diseases [10,11].
2. Sleep: Sleep disorders have implications for daily life, including fatigue, low performance, and difficulties to complete professional, family or social obligations. There is also a correlation between sleep disorders and neurological disorders [12]. Even in the absence of sleep disorders, the amount and quality of sleep have a major impact on brain health, cognitive function, and mental wellbeing.
3. Nutrition: How much we eat and what we eat represent an important pillar for brain health. An unbalanced diet can result in a lack of nutrients, which can have a deep impact on our overall health. In addition, nutritional factors have been linked to diseases such as dementia or Alzheimer’s disease [10]. A balanced Mediterranean diet can impact cognitive function, and certain nutritional supplements might have an effect on mood, motivation, and initiative. Furthermore, the body mass index (BMI) appears to correlate with mental wellbeing and cognitive abilities [13,14].
4. Cognitive activity: As we get older, our brains require less strain to perform everyday activities. However, our brain needs to face new challenges in order to stay healthy. It is as important to “exercise” our mind as it is to exercise our bodies. Cognitive impairment can be the result of neurological diseases such as Alzheimer’s disease or Parkinson’s disease. Keeping an active brain can preserve brain plasticity and promote brain resilience and cognitive reserve. Cognitive activity can, but does not necessarily have to, involve computer-supported cognitive training [15].
5. Vital plan: Meaning in life and life purpose are the focus of many psychology studies from the last decades of 20th century [16,17,18] and alterations or lack of a defined vital plan are associated with many disorders like anxiety, depression, or even mortality. These disorders are known to interfere in brain health [19]. Our human brain has a property that animals lack: It allows us to project ourselves into the future. Prospecting, the ability to imagine what it will be like to try to make a goal or a dream into a reality, is an essential function for our brain and we need to encourage it by defining a vital plan, a purpose in life that transcends us as individuals. This is so important that it seems to mediate the effect of all other pillars onto our brain health.
6. Social interactions: We are social beings and our brain needs relationships. The time spent with family and friends or getting to know and relating to our neighbors and colleagues is important. Loneliness is not only bad for brain health, it is a deadly disease. Individuals with a high number of social interactions experience significantly less cognitive decline compared to those who are lonely or isolated [20,21]. It also has been shown that social interactions and environment can help to improve brain plasticity after a brain lesion [22].
7. Overall health: Overall health is an important factor due to the existing strong relations between overall health and brain health. For example, there is a close link between chronic diseases and depression [23,24] and systemic diseases, such as diabetes or hypertension, pose critical risks for brain health. Therefore, we should have check-ups, go to the doctor regularly, follow their recommendations, and pay attention to the conditions and diseases we have. However, we now also know that the opposite direction is also important, good mental and brain health promote overall health and wellbeing.

Monitoring all these pillars that contribute to brain health is the key (1) to identify the different factors that may have an impact on brain health in a given individual, and (2) to design effective personalized interventions to prevent the onset of cognitive decline and sustain mental wellbeing.

Monitoring technologies are having a great boom nowadays. We can find a wide range of devices and sensors to track different parameters [25]. These are small and wireless, and can be taken everywhere without feeling uncomfortable. Examples of these are wearables, smartphones, and other similar devices, which can collect data about ourselves [25]. The questions that come up are: “Are these data enough to model our daily habits?”, “is it possible to create a semi-automatic multi-domain intervention system that modifies our habits?” and “can technology help us to maintain, or even improve, our brain health?”.

The aim of this study is to carry out a systematic review of the literature analyzing what technologies have been used to monitor daily lifestyle parameters and which ones could best help to improve or modify people’s habits in multimodal interventions for brain health promotion. A special focus was placed on the goals and results obtained by said technologies, as well as on identifying the target populations used to validate their use.

We realize that in focusing only on peer-reviewed publications, we capture a small portion of the exciting developments and relevant solutions, since much pertinent work has been and is being done by app developers and commercial ventures. Eventually, an analysis of that landscape and knowledge would be very valuable.

## 2. Materials and Methods

**Figure 1:**
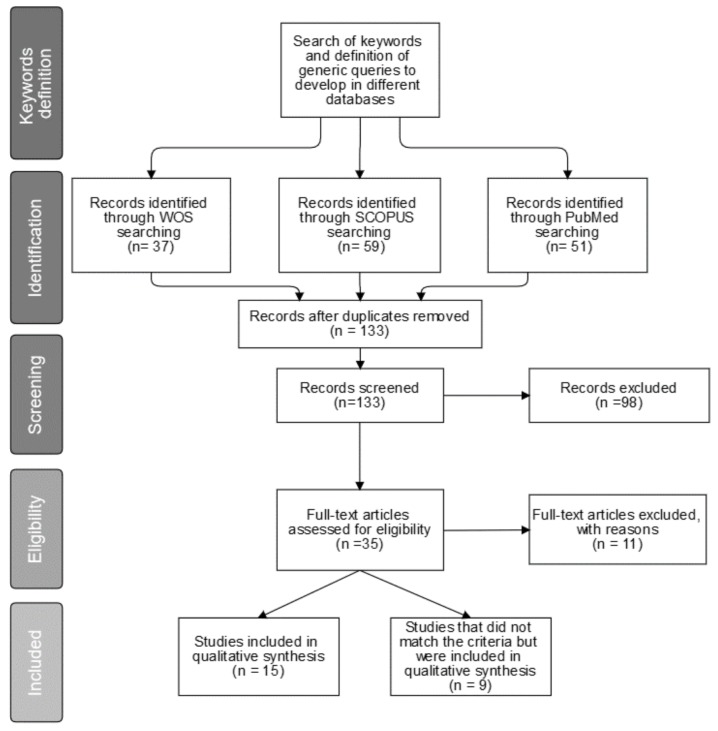
Study selection according to the Preferred Reporting Items for Systematic Reviews and Meta-Analyses (PRISMA) statement.

Figure 1 shows the different phases defined in our methodology according to the guidelines set out by the Preferred Reporting Items for Systematic Reviews and Meta-Analyses (PRISMA) statement [26]. 

### 2.1. Keywords Definition

To facilitate the process of identifying the keywords, we defined five categories to work with:

- General: Terms that define the main field of the study. In this case, terms related to brain health or cognitive functioning, including cognitive deterioration and cognitive reserve. The terms ‘brain health’ and ‘cognitive’ (which include terms above and more) were chosen.
- Associated: Terms associated with the topic. In this case, terms associated with cognitive decline (e.g., age, aging).
- Pillars: Terms that are associated with the specific pillars of intervention (as defined in [3]) identified as critical variables that affect brain health. (e.g., nutrition, sleep or socialization).
- Techniques: Terms that are often used in projects related to interventions and monitoring of daily life activities (e.g., intervention, monitoring, adherence, etc.).
- Technologies: Technical terms that usually appear in studies related to eHealth and telemedicine (e.g., wearable, eHealth, ICT, etc.).

The complete list of categorized keywords is provided in Table 1.

### 2.2. Identification

Three databases were selected for this review. Searches were conducted in Scopus, Web of Science (WOS) and PubMed. We associated terms within a given category with a logic OR operator. We linked queries between categories with a logic AND operator. This way we ensured that all categories were examined, and that at least one term for each category applied to any identified publications. A complete table summarizing the query employed can be found in Appendix A.

Several restrictions were applied at this stage. First, articles had to include a cohort of subjects and/or feature a controlled study. Reviews, studies about other topics (e.g., surgery) and studies focused on very specific illnesses or medical conditions (e.g., strokes) were excluded. A temporal restriction was applied to cover a date range of five years, between 1 January 2013 and 19 May 2019. Finally, duplicate studies were removed.

### 2.3. Screening & Eligibility

During the screening phase, the title and abstract of the studies were scanned. Following a similar methodology to the review carried out by Vegesna et al. [27], we checked whether identified publications matched the following rules defined in the criteria based on the population, intervention, comparator, outcomes, timeframe, and study design (PICOTS) format [28]:

- Population: Participants had to be at least 18 years old as we aimed to focus on adults and exclude pediatric populations. Participants had to be healthy and thus could not be diagnosed with any particular disease or disability.
- Intervention: The intervention should not be related to one particular disease (e.g., Alzheimer’s disease or multiple sclerosis) or to any single particular ability or problem (e.g., driving). Rather, the intervention should be focused on habit improvement and daily life monitoring. It also must involve the use of at least one of the following technologies: (1) Web application (2) mobile phone (3) wearables (4) biosensors (5) medical devices (e.g., fMRI) (6) computer tasks.
- Comparator: Both placebo and active interventions were taken into consideration.
- Outcome: The outcome must be referred to core aspects of brain health or cognitive function, including (but not exclusively) one or more of the seven pillars (e.g., sleep or physical activity). Ideally, the publications should also contain an outcome of therapy adherence or usability.
- Timeframe: Both short- and long-term outcomes were taken into account.
- Study: Studies could be either randomized controlled trials (RCTs) or observational studies. Protocols, systematic reviews, nonsystematic reviews, case studies, commentaries, and letters or editorials were excluded.

During the eligibility phase, the full document of the identified studies was analyzed. The same exclusion criteria as in the screening phase were applied once more. 

### 2.4. Included

The resulting studies were classified according to different criteria. The first one referred to the way in which monitoring had taken place, according to:

- Heavy monitoring: When people needed a hospital or a controlled site to do specific tasks or specific tests.
- Medium monitoring: When participants were monitored using smartphones, wearables or biosensors that are not intrusive.
- Light monitoring: When participants were only monitored using questionnaires and tests or providing self-report data, through web or mobile applications.
- No monitoring: When no monitoring took place or is not reported.
- Studies were also subcategorized according to how the intervention was carried out. We defined two categories:
- Dynamic intervention: When the intervention was adaptive and could change to fit the participant’s behavior patterns and evolution.
- Static intervention: When the intervention was the same for all participants, based on pre-specified criteria and rules, and was not modified throughout the study.

Finally, third subcategorization was performed according to how the technology was provided to the users: (1) Web application, (2) mobile phone, (3) wearables, (4) biosensors, (5) medical devices (fMRI, etc) or (6) computer tasks.

## 3. Results and Discussion

Figure 1 shows the search results. A total of 147 studies were found amongst the three databases (37 results from Web of Science, 59 from SCOPUS and 51 from PubMed). After removing duplicates, there were 133 studies left. During the first screening phase, 98 publications were discarded because they did not match the selection criteria. After that, the second screening phase took place. Although 20 studies did not fit the criteria, nine of them were included in the qualitative synthesis because they were considered relevant for the discussion and conclusions. A total of 15 studies met the inclusion criteria. These selected papers and the nine additional publications discussed are listed in Table 2 and Table 3, respectively.

The low number of only 15 studies that met all our criteria was unexpected. We hypothesize that the number of studies would be much higher had we considered monitoring and interventions for specific illness because much of the current research focus remains centered around the reduction of disability in patients with established diagnoses, rather than around the promotion of brain health and prevention of illness. Furthermore, as mentioned in the introduction, we believe that a large portion of the relevant efforts is taking place as part of commercial ventures and other initiatives that do not necessarily get disseminated via peer-reviewed publications. 

### 3.1. Distribution on Pillars

**Figure 2:**
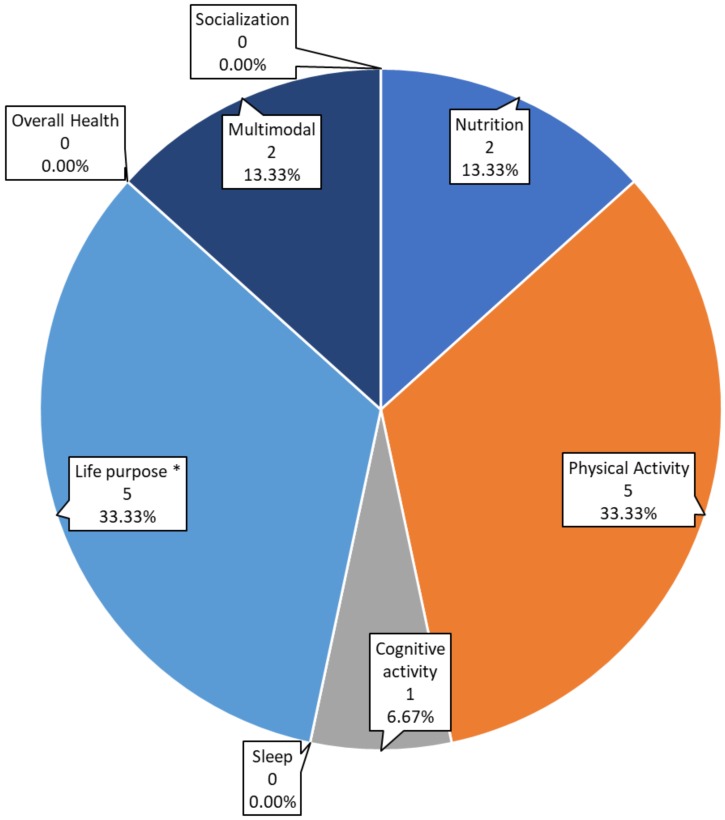
Number and percentage of studies focused on each pillar. *Life purpose includes studies focused on behavior and cognitive behavioral therapy.

All the studies included in the review focused on one or two of the defined pillars. Studies on cognitive behavioral therapy (CBT) and habit management have been included in the “life purpose” pillar. As can be seen in Figure 2, the five studies focused on physical activity [27,32,34,36,37] represent a third of the total, and the five focused on life purpose studies [28,30,35,39,40] represent another third. Four of the studies related to the “life purpose” pillar [30,32,41,42] (80% of the “life purpose” pillar related studies and 26.66% of the total of studies) focus on habit management and CBT. Only two of the studies [36,40], which represent 13.33%, are multimodal. The study from Merriman et al. [36] combined physical exercise and cognitive activity, whereas the one from Rodrigues et al. [40] focused on nutrition and physical exercise. There are no studies focused on sleep, overall health, or socialization that otherwise fulfilled our filtering criteria.

Our results reveal that there are pillars which to-date have rarely or not been studied from a brain health perspective. Clearly there is a lot of work pending on these areas, including sleep, socialization, cognitive activity, and nutrition. Furthermore, it is striking that very few studies are multimodal, even though multi-pronged approaches are likely to be essential in the promotion of overall brain health. 

### 3.2. Monitoring

**Figure 3:**
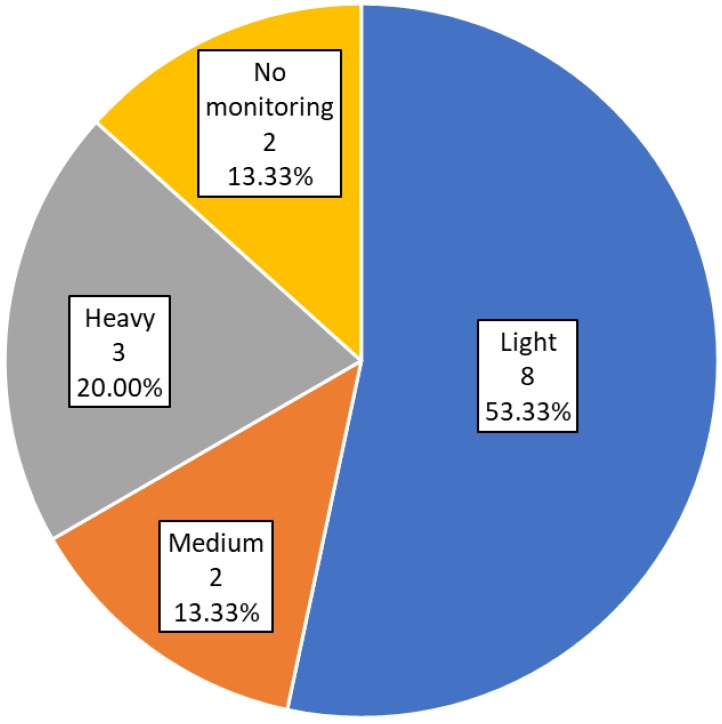
Monitoring results. Number and percentage of studies in each category.

Figure 3 shows the classification of studies according to how they monitor subjects. Eight of the studies identified (53.33%) [30,31,32,33,35,37,40,41] employed light monitoring, based on questionnaires and tests to get data and parameters from users. Three of the studies [29,36,39] (20%) employed heavy monitoring with controlled spaces or specific devices. Two studies [34,42] (13.33%) focused on medium monitoring, based on smartphones, wearables, and sensors. The remaining two studies [38,43] (13.33%) did not use any kind of monitoring systems, and relied instead on baseline and post-intervention assessments. 

Light monitoring questionnaires and user self-reported data are certainly easy to implement and can gather relevant and accurate information about the users. Nonetheless, the recording (possibly in addition to such subjective reporting) of objective data derived from sensors or wearables seems important. To date, this has been quite rarely implemented. Nonetheless, we expect that the use of such technologies steeply grows in the near future. The use of controlled environments with specific tools like the special workstations of the study by Commissaris et al. [29] or the use of virtual reality in the study by Merriman et al. [36] can be particularly valuable to test and improve the use of new technologies or to carry out proof of concept trials. However, such approaches are not well suited for long interventions and fail to capture a true reflection of daily life.

### 3.3. Intervention Style

**Figure 4:**
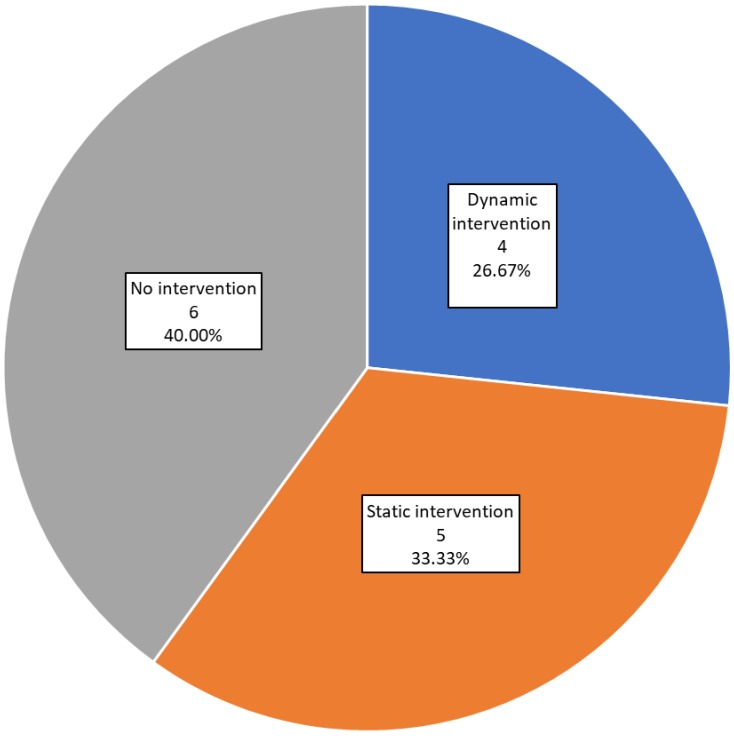
Intervention results. Number and percentage of studies in each category.

Figure 4 shows the distribution of studies according to the type of intervention. Six of the studies [29,33,36,38,40,43] (40%) did not carry out any kind of intervention. Static interventions were conducted in five of the studies [31,34,35,41,42] (33.33%). The remaining four studies [30,32,37,39] (26.67%) report a dynamic automatized or semi-automatized guided intervention. 

There is no doubt that deploying interventions is challenging and thus, it is not surprising that most studies to date have monitored lifestyles, but often not deployed interventions. Studies focused on monitoring and data analysis are obviously critical to establish reliable metrics and develop behavior and parameter models that can ultimately predict and characterize the manifestation of brain diseases, and eventually, assess the efficacy of interventions. Studies that apply static interventions do not apply intelligence or other algorithms of any kind, and their interventions are not personalized or adapted to the user. The ultimate goal ought to be to develop such personalized interventions supported by predictive algorithms, but that is obviously most challenging and ideally implemented on the foundation of defined models or previous data. Only 26,67% of the studies included neuronal networks, deep learning, machine learning or clustering approaches to inform more dynamic, adaptive, and personalized interventions. We expected a larger number of studies applying artificial intelligence approaches both on intervention and personalization, and expect that future developments will do so.

### 3.4. Technology Used

**Figure 5:**
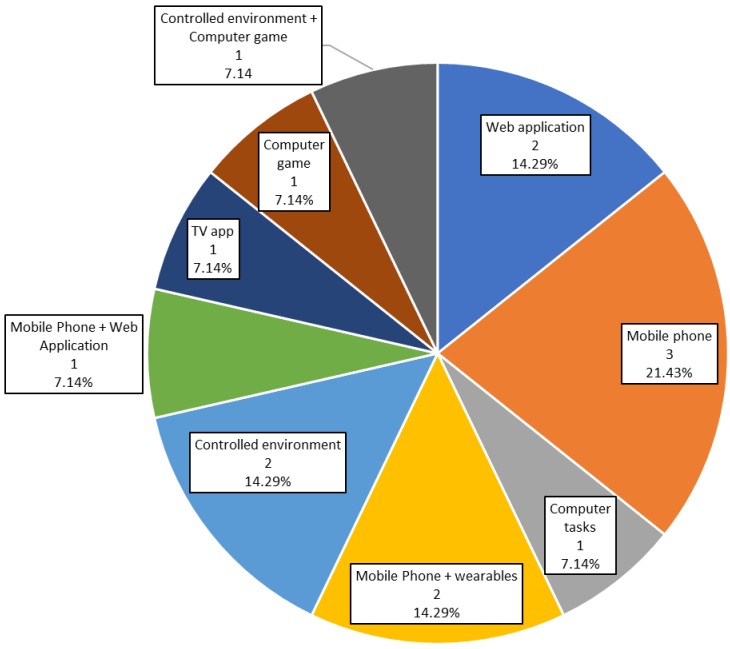
Technologies results. Percentage of studies in each category.

The distribution of studies according to the technology used is summarized in Figure 5. Six of the included studies (42.86%) used mobile phones as a technological solution. They are used on their own in three studies (21.43%) [31,32,43] or in combination with wearables in two studies (14.29%) [34,42] or web applications in only one study (7.14%) [37]. For example, Wirth et al. [31] used mobile phones to report monitoring data via telephone. Pavel et al. [32] study used mobile phones for coaching via telephone and email.

Web applications were used in three studies (21.43%). In two of them [30,35] (14.29%) web applications were the only technological solution employed, whereas in the other (7.14%) they were completed by mobile phones [37].

Three studies (21.43%) developed a controlled environment. For example, Commissaris et al. [29] and Konstantinidis et al. [39] used a controlled environment and gamification techniques, whereas Merriman et al. [36] implemented a serious game. Konstantinidis et al. [39] and Merriman et al. [36] employed a Wii Balance Board, which is a specific gaming hardware solution.

Finally, there are single studies of specific technical solutions. For example, Zielhorst et al. [41] implemented a videogame to improve CBT. The study by Ramnath et al. [33] employed computer tasks to measure various cognitive variables and physical tasks to measure physical status based on questionnaires. Finally, the study reported by Rodrigues et al. [40] used a smart TV application.

Thus, in the reported peer-reviewed literature to date, mobile phones are the most commonly used tool, but usually, it is not exploited to the fullest. Wirth et al. [31] used mobile phones to collect data via a phone call, ultimately an app might be more reliable, more usable, and potentially less expensive.

Worth highlighting is also the study by Rodrigues et al. [40], which employed a smart TV application. This is an interesting approach, considering that smart TVs are becoming very popular, and could also be a useful tool for coaching and/or monitoring. This is especially true for studies that focus on elder participants who are not very used to manage mobile phones or other technological devices and usually have vision problems which could be overcome with a big TV screen.

Games are common in the studies published to date. Two studies (14.29%) use games, and many of the other studies apply gamification techniques. Without a doubt, gamification is appealing and likely can contribute to increasing study adherence. Ultimately personalization of the gamification features may be worth exploring given difference preferences by different individuals.

It is surprising that only two studies to date have been published using wearables (only 14.29%). Nowadays smartphones or tablets are more frequently used than computers [44]. Moreover, almost every task can be implemented in an app or web format thus making it more accessible. There is little doubt that the use of mobile technologies, wearables, and apps will increase rapidly in future studies.

### 3.5. Technologies Related to Pillars

**Figure 6:**
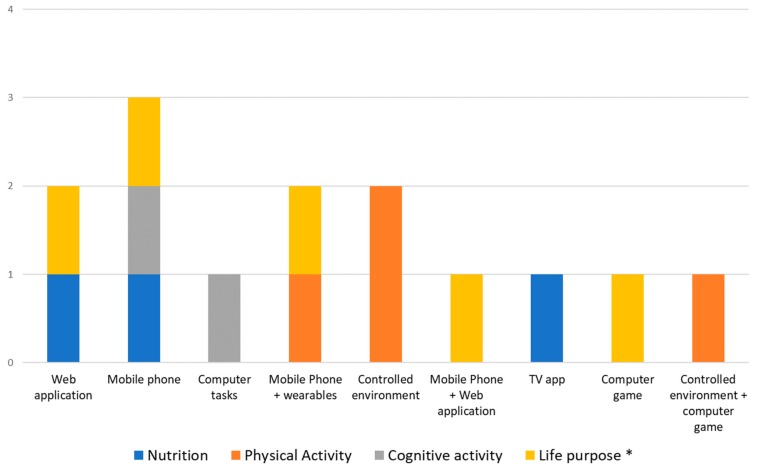
Technologies applied for monitoring and intervention on each pillar. *Life purpose includes studies focused on behavior and behavioral changing.

Figure 6 shows the distribution of studies according to each of the seven pillars hypothesized to support brain health. In fact, in the studies identified, only four pillars were monitored. Physical exercise was monitored via controlled environments in three studies [29,36,39] and with mobile phone and wearable devices in one [34]. Nutrition monitoring and interventions are based on applications. We can find a mobile phone application in one study [31], a web application in another [35], and a smart TV application in a third [40]. Cognitive activity is measured with computer tasks in one study [33], and with a mobile phone in another [43]. Finally, for monitoring of life purpose, in which we have included CBT and habits management studies, there has been the greatest number of different technological solutions tried: One of the studies used a web application [30], another used mobile phones [32], the third used mobile phones and wearable devices [42], the fourth used a web application and a mobile phone application [37], and the last one used a computer game [41]. 

Consideration of these results together with the previous ones about monitoring and intervention, reveals several interesting facts:

- The vast majority (75%) of studies related to physical exercise focus on proofs of concept and use specific controlled environments, where they integrate or replicate the sensors that could be found in a wearable device. Future studies, therefore, are likely to employ wearables to capture similar outcomes.
- Nutrition is difficult to monitor with sensors, so it is usual to find that both, monitoring and intervention, are carried out with questionnaires and guidelines. This is why web and mobile applications are the most used (75% of them).
- Surprisingly, the same occurs with the cognitive pillar, where only tasks or questionnaires are used. Future studies ought to leverage mobile trackers, wearables, and phones to try to capture relevant information regarding cognitive function in a real-life setting and employing passive, non-intrusive designs.
- Although there exist some non-intrusive devices to measure brain signals (mainly EEG), these are not yet comfortable, portable or reliable enough to use in daily life tasks and in long periods.

### 3.6. Demographic Data

**Figure 7:**
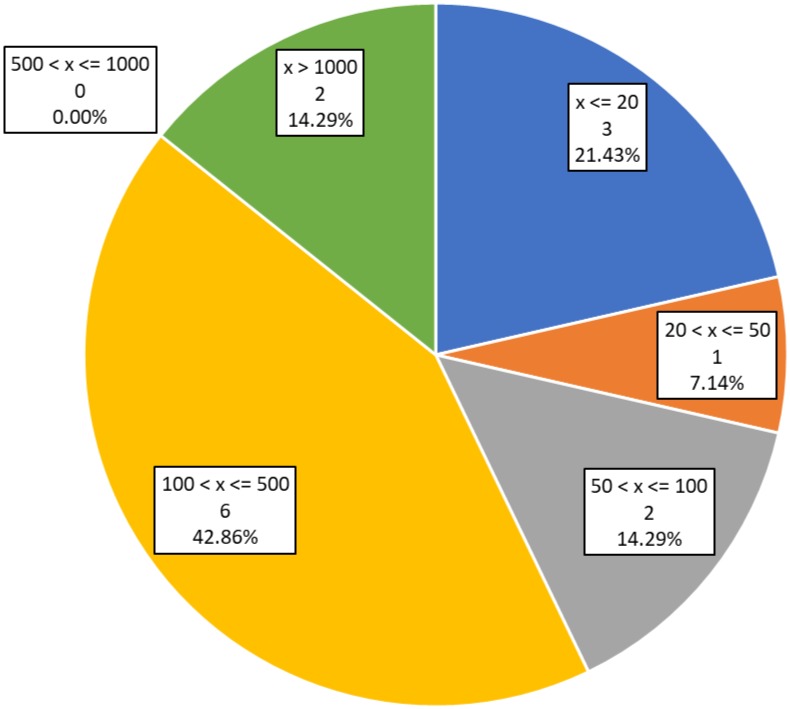
Number and percentage of studies on each sample size range. The “x” represents the study sample size.

Figure 7 presents the sample size of subjects involved in validation for each study. Three studies [29,30,35] (21.43%) report a sample lower or equal to 20 people.

One study [43] (7.14%) reports a sample size between 20 and 50 people. Two studies [33,36] (14.29%) have a sample from 50 to 100 users. Six of the studies analyzed [31,32,37,39,40,41] (42.86%) on this review report a sample size between 100 and 500 users. Another two studies (14.29%) have a sample size larger than 1000 people. Two studies, the one by Lange et al. [35] and the one by Veronese et al. [38] employed sample sizes of over 3000 people. Note that one study included in this analysis [42] does not report a sample size.

Results from studies with small samples (e.g., <50 participants) must be taken with prudence, and usually, further investigation will be needed to confirm findings. With such small sample sizes, the possibilities of unexpected selected bias and limited extrapolation of the findings are quite high. Even with sample sizes over 100 people, there are a lot of potentially relevant demographic variables that can introduce bias and thus limit the broad relevance of the results.

**Figure 8:**
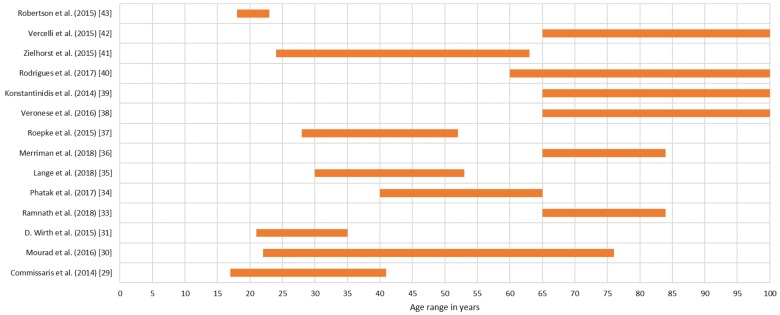
Age range of each study.

Figure 8 shows the distribution of studies according to the age of the validation cohorts. Six studies [33,36,38,39,40,42] (42.86%) were focused on elder people. Four studies [29,34,35,37] (28.57%) focused on middle-aged adults, and two studies [31,43] (14.29%) were focused on young participants. The study by Pavel et al. [32] does not report the age group of its participants. The study by Mourad et al. [30] and the study by Zielhorst et al. [41] included a wide range of ages, but the latter excluded individuals older than 63 years.

Studies contrasting across age ranges are still lacking and yet will be important particularly given the large differences in familiarity and levels of comfort with technology in younger generations as contrasted with the older ones.

We can see in Table 2 that eight of the analyzed studies were carried out, totally or partially, in Europe (57.14%). Only two studies (14.28%) were carried out globally. Brain health is a global problem and cultural differences are likely relevant factors. Therefore, a greater number of cross-cultural or global studies are critical. This is particularly the case in the assessment of technologies given likely cultural differences in their adoption.

### 3.7. Correlation between Lifestyle Habits Factors and Brain Health

The selected papers barely report data to extract conclusions about how intervention and monitoring correlate with brain health improvement. Only nine studies [31,32,34,35,36,38,39,41,43] report positive results, which implies significant better results from test group than control group. Four studies [29,30,33,37] report neutral results, which implies that there are no significant differences between the control and test groups. Finally, two studies [40,42] do not report results in this direction.

Not all the studies had the same focus and hypothesis, and not all the studies used the same evaluation methods. Due to this heterogeneous group of studies, and because of the lack of several details in many of them, it is difficult to extract a solid correlation between habit improvement and its impact on brain health. 

### 3.8. Limitations and Out of Criteria Studies

We realize that our review has a number of limitations. This work is focused on BBHI pillars, and thus we conducted a very focused search in brain health studies addressing them. As a result, we obtained a small sample of studies. There are many relevant developments, for example, in commercial ventures and companies, which are not captured in peer-reviewed academic publications. We also know that there are many other studies, for example, conducted on diverse patient populations, which use technologies and techniques that are relevant and valid for the monitoring and intervention of brain health factors. These are some of the reasons why we also analyzed studies, for discussion purposes, that had been initially discarded because they did not fit our strict criteria, yet had valid resources for monitoring or brain health interventions. These studies are summarized in Table 3. The study by Robert et al. [45] is focused on physical exercise in patients with Alzheimer’s disease. Robert et al. developed an interesting tracking system based on cameras and an intelligent room to do daily life activities, and shoe motion sensors to track movement in outdoors activities. Chen et al. [46] used faceLab 4 [54] to get different parameters like pupil dilation and position to try to characterize emotions and cognitive load. Although the results were not positive, it is an interesting concept and could be an effective tool to measure parameters related to brain health, especially those related to the vital plan and cognitive pillars. Cerasa et al. [47], Baglio et al. [48] and Cerasa et al. [51] studied patients with Parkinson’s disease and Alzheimer’s disease respectively. Both of them used medical imaging, functional Magnetic Resonance Image (fMRI), with cognitive tasks or questionnaires to analyze brain activation, and they reported positive results. The study by Baglio et al. [48] applied a multimodal intervention, which is an interesting and promising tendency given our growing understanding that different factors coming together are critical for brain health. Manzoni et al. [49] implemented virtual reality (VR) to improve a CBT program for obese people and change their habits to healthier ones. It reports positive results with this approach.

The study by Mehrabian et al. [50] is interesting because it focused not only on patients but also on caregivers. It employed web applications and interviews. Such approaches could be meaningfully extended to capture social interactions more fully. The study by Evensen et al. [52] developed a mobile application reinforced with accelerometers to monitor physical exercise in patients admitted in the hospital with positive results. The study by Hacker et al. [53] used avatars for personalizing CBT to get better adherence. All these studies suggest that techniques and tools developed for other uses can be also and meaningfully applied to monitor and intervene to promote healthy habits for maintenance and improvement of brain health.

Even with our limitations, it seems clear that technology to monitor and intervene to promote brain health is a growing topic. Our hypothesis is that investigations in this topic will provide large amounts of information which may lead to a real transformation in how we understand the human brain in the near future. With this analysis as a starting point, we plan to contribute to this field with the Intelligent Brain Coaching project (Spanish National Project. Programa RETOS: DPI2017-86088-C3-1-R.) This project will focus on analyzing data from the cohort of volunteers recruited into the Barcelona Brain Health Initiative [2], create brain health models and develop new ways of monitoring and coaching applied to brain health. We hope we can create semi-automatic coaching systems that can provide a new tool to promote improvement in brain health and contribute to the fight against neurodegenerative diseases.

## 4. Conclusions

Our study highlights the fact that nowadays we are not extracting all the potential that we could obtain from the application of technologies for monitoring brain health. Although this study has some limitations because of the specific topic of search, based on BBHI hypothesis, results have shown that in most of the cases, we are only migrating old tests and recipes to a digital format. We are not exploring the wide amount of data that these new devices offer us. We are not looking for new relations or new parameters that could give us new knowledge and new ways of preventing or healing neurological diseases.

Multi-modal approach to brain health issue is not a tendency right now, although brain health has been proved to be not related only to one single cause. If we want to tackle the problem, it seems logical that it is needed to intervene in all causes, in all domains, in a coordinated way. As important as this, adapting the intervention to each person is crucial., and we have not found studies focused on that. There are multiple causes and they do not affect each person equally, so intervention should be adapted to the special circumstances of each one.

The use of artificial intelligence, and many other techniques that are largely known in other fields can be the inflection point to get new ways of monitoring and intervention applied to brain health. With the impulse of the modern artificial intelligence like deep learning, we could find new factors and parameters relations not detected yet [55]. The use of new devices that can enable us to continue monitoring daily life activities. However, today we are not applying any of them. This tendency should change in a few years as it has changed in other fields, like commercial, advertising or fitness. With the potential that actual technology offers could revolutionize the health field. Nowadays users have already integrated, mainly thanks to the era of mobile phones and wearables, many commercial devices or solutions. Although they have been originally conceived to target other populations, we foresee that only those technological solutions able to easily integrate already existing monitoring devices and wearables will succeed at a global level beyond pure research.

## Figures and Tables

**Table 1:** Search terms and categories. Terms appear separated by semicolons.

Categories	Terms
General	Brain Health; Cognitive
Associated	Older Adults; Aging; Ageing; Elderly; Geriatrics; Young elders; Aged; Older Person
Pillars	Nutrition; Physical exercise; Cognition; Social; Purpose in life; General health; Diet; Physical activity; Cognitive activity; Socialization; Psychological wellbeing; Comprehensive health; Physical; Cognitive; Cognitive training; Vital plan; Mindfulness; Rest; Sleep; Sleeping; Relax; Global health
Techniques	Exercise; Coach; Intervention; Coaching; Treatment; Monitoring; Adherence; Motivation
Technologies	Wearable; Computer; ICT ^1^; Machine learning; Data mining; RMT ^2^; Data mining; Artificial intelligence; Deep Learning; eHealth; mHealth; Biosensor; Neuronal network; Predictor; Mobile; Smartphone; Technology

^1^ Information and Communication Technologies ^2^ Remote Monitoring Techniques.

**Table 2:** Selected papers.

First Author, Year	Region	Variable	Study Design/Study Duration	Methodology and Technologies	Sample Size	Age Groups	Feedback Loop/End-User	Results	Funding Body
Commissaris et al. (2014) [29]	Netherlands and Germany	Physical exercise	Cohort/1 working day (7–8 h)	Heart Rate Monitor/3D kinematics measurement system	15	29 years (SD 12)	Office Workers and Employers	Neutral	German Social Accident Insurance (DGUV)
Mourad et al. (2016) [30]	Sweden	Life purpose ^1^	RCT/4 weeks	Internet-delivered program with questionnaires	15	22–76	Self/User	Neutral	County Council of Östergötland/Medical Research of Southeast Sweden
D. Wirth et al. (2015) [31]	South Carolina (USA)	Nutrition	Cohort/14 days	Phone questionnaires	430	21–35	NR	Positive	Coca Cola Company
Pavel et al. (2016) [32]	NR	Life purpose ^2^	RCT/25 weeks	Mobile phone	204	NR	Self/User	Positive	NR
Ramnath et al. (2018) [33]	South Africa	Physical exercise and cognitive activity	Cohort/1 session	Questionnaires & physical tasks	70	65–84	Self/User	Neutral	NR
Phatak et al. (2017) [34]	United States	Physical exercise	Cohort/14 weeks	Fitbit Zip/Mobile App/Personalization	20	40–65	Self/User	Positive	National Science Foundation
Lange et al. (2018) [35]	Germany	Nutrition	Cohort/2 years	Web App	3000	41,5 (SD 11.9)	Self/User	Positive	German Ministry of Education and Research
Merriman et al. (2018) [36]	Ireland	Physical exercise	RCT/5 weeks	PC game/Wii Balance Board/Gamification/Serious Game	70	65–84	Self/User	Positive	European Commission Seventh Framework Programme ‘VERVE’ Project and by Principal Investigator award and TIDA award to FNN from Science Foundation Ireland
Roepke et al. (2015) [37]	World	Life purpose ^3^	RCT/6 weeks	Smartphone-Based/Internet-Based Self-Help Tool	283	40.15 (SD 12.4)	Self/User	Neutral	Private donation
Veronese et al. (2016) [38]	Italy	Physical exercise	Cohort/4.4 years	Data Analysis	3099	>65	NR	Positive	Fondazione Cassa di Risparmio di Padova e Rovigo/University of Padova/Azienda Unità Locale Socio Sanitaria
Konstantinidis et al. (2014) [39]	Europe	Physical exercise	Cohort/7–8 weeks	Serious Game/Computer application/Data analysis/Exergaming/Wii Balance Board	116	>65	Self/User	Positive	European Union
Rodrigues et al. (2017) [40]	Portugal	Nutrition and Physical exercise	RCT/6 months	TV app	282	>60	Self/User	NR	European Economic Area
Zielhorst et al. (2015) [41]	Netherlands	Life purpose ^4^	Cohort/10–15 days	CBT/Gamification	101	24–63	Self/User	Positive	NR
Vercelli et al. (2017) [42]	Europe, Australia, and Asia	Life purpose ^5^	NR	Smartphone app/wearables	NR	>65	Self/User	NR	European Union
Robertson et al. (2015) [43]	United States	Cognitive activity	RCT/1 h	Mobile app/Motion sensors/Real Time Annotation Tool	42	19.88	Self/User	Positive	National Science Foundation

^1^ Emotions and habits; ^2^ Habits; ^3^ Depression; ^4^ Stress and habits; ^5^ Habits and daily life monitoring.

**Table 3:** Out of criteria included papers.

First Author, Year	Region	Variable	Study Design/Study Duration	Methodology and Technologies	Sample Size	Age Groups	Feedback Loop/End-User	Results	Funding Body	Exclusion
Robert et al. (2013) [45]	France and Taiwan	Physical exercise	Cohort/1 day	Intelligent room (2D video camera, ambiance microphone, motion sensor, and tri-axial accelerometer mounted on the shoes)	64	>65	Therapist	Positive	Innovation Alzheimer and ARMEP associations	Alzheimer
Chen et al. (2013) [46]	Australia	Cognitive activity	Cohort/1 session	FaceLAB for pupil dilation and position	15	20–48	Therapist	Negative	Australian Government	No Brain Health
Cerasa et al. (2014) [47]	Italy	Cognitive activity	RCT/6 weeks	RehaCom (Cognitive training tasks), 3T Scanner for images	20	61.1 (12.4 SD)	Therapist	Positive	Ministerio Univesita’ e Ricerca	Parkinson
Baglio et al. (2015) [48]	Italy	Stress. Multidisciplinary intervention	RCT/32 Weeks	fMRI and questionnaires	60	65–85	Therapist	Positive	Ricerca Corrente (Italian Ministry of Health)	Alzheimer
Manzoni et al. (2016) [49]	Italy	Habits	RCT/11 weeks	Virtual Reality/CBT	158	18–50	Self/Patient	Positive	NR	Obese people
Mehrabian et al. (2018) [50]	France	Intervention	Cohort/40 min	Interviews + web app	92	54–85	Patient/Caregiver	Positive	National Research Agency and the Foundation Mederic Alzheimer	Cognitively impaired/caregivers
Cerasa et al. (2013) [51]	Italy	Cognitive Function	RCT/6 weeks	fMRI/cognitive computerized tasks	26	32 (SD 10)	Clinicians	Positive	Fondazione Italiana Sclerosi Multipla onlus and Ministero Universita’ e Ricerca	Multiple sclerosis
Evensen et al. (2017) [52]	Norway	Physical Activity	Cohort/3 months	accelerometers/activePal	38	82.9 (SD 6.3)	Clinicians	Positive	Liaison Committee between the Central Norway Regional Health Authority and the Norwegian University of Science and Technology	Hospitalized patients
Hacker et al. (2015) [53]	USA	Personalization	Cohort/4, 20 days	Web application	176	11 to 15	Self/User	Positive	National Science Foundation	Not health-oriented

## Appendix A

Table A1 shows the successive phases of the query formation. Subqueries 1 to 5 conform the aggrupation of different categories. Then a filter of study type was added on subquery 6. Exclusions were included in subquery 7. The final subquery, index 8, is the conjunction of all the previous subqueries.

**Table A1:** Indexed query formation process.

Index	Query	Category
1	( “brain health” OR “cognitive” )	General Terms
2	( “brain health” OR “cognitive” OR “young elders” OR “aging” OR “older adults” OR “ageing” OR “elderly” OR “aged” OR “older person” OR “geriatrics” )	Associated Terms
3	( “nutrition” OR “diet” OR “physical” OR “physical exercise” OR “physical activity” OR “cognitive” OR “cognition” OR “cognitive activity” OR “cognitive training” OR “social” OR “socialization” OR “vital plan” OR “purpose in life” OR “psychological wellbeing” OR “mindfulness” OR “general health” OR “comprehensive health” OR “global health” OR “sleep” OR “sleeping” OR “relax” OR “rest”)	Pillar related Terms
4	( “adherence” OR “motivation” OR “monitoring” OR “coaching” OR “coach” OR “treatment” OR “intervention” OR “exercise” )	Technique related terms
5	( “smartphone” OR “mobile” OR “ICT” OR “RMT”OR “mHealth” OR “eHealth” OR “data mining” OR “predictor” OR “machine learning” OR “deep learning” OR “neuronal network” OR “artificial intelligence” OR “computer” OR “biosensor” OR “wearable” OR “technology” OR “technologies”)	Technology related terms
6	( “observational study” OR “controlled study” )	Study filter
7	NOT ( “schizophrenia” ) AND NOT ( “cancer” ) AND NOT ( “pediatrics” ) AND NOT ( “epilepsy” ) AND NOT ( “drugs” ) AND NOT ( “diabetes” ) AND NOT ( “stroke” ) AND NOT ( “dementia” ) AND NOT ( “transplant” ) AND NOT ( “fracture” ) AND NOT ( “traumatic” ) AND NOT ( “surgical” ) AND NOT ( “EEG” ) AND NOT ( “disorder” )	Exclusions
8	[1] AND [2] AND [3] AND [4] AND [5] AND [6] AND [7]	Resultant query
